# Temporal Characteristics of the Chinese Aviation Network and their Effects on the Spread of Infectious Diseases

**DOI:** 10.1038/s41598-017-01380-5

**Published:** 2017-04-28

**Authors:** Jianhong Mou, Chuchu Liu, Saran Chen, Ge Huang, Xin Lu

**Affiliations:** 1 College of Information System and Management, National University of Defense Technology, Changsha, 410073 China; 2 School of Mathematics and Big Data, Foshan University, Foshan, 528000 China; 3 Department of Public Health Sciences, Karolinska Institutet, Stockholm, 17 177 Sweden; 4 Flowminder Foundation, Stockholm, 11 355 Sweden

## Abstract

Aviation transportation systems have developed rapidly in recent years and have become a focus for research on the modeling of epidemics. However, despite the number of studies on aggregated topological structures and their effects on the spread of disease, the temporal sequence of flights that connect different airports have not been examined. In this study, to analyze the temporal pattern of the Chinese Aviation Network (CAN), we obtain a time series of topological statistics through sliding the temporal CAN with an hourly time window. In addition, we build two types of Susceptible-Infectious (SI) spreading models to study the effects of linking sequence and temporal duration on the spread of diseases. The results reveal that the absence of links formed by flights without alternatives at dawn and night causes a significant decrease in the centralization of the network. The temporal sparsity of linking sequence slows down the spread of disease on CAN, and the duration of flights intensifies the sensitiveness of CAN to targeted infection. The results are of great significance for further understanding of the aviation network and the dynamic process, such as the propagation of delay.

## Introduction

Aviation transportation systems have developed rapidly in recent years^1^. According to the International Civil Aviation Organization (ICAO), the number of airlines in the world increased by approximately 30% between 2005 and 2014^2^. By 2014, there were 1,397 commercial air companies, 3,864 airports and 49,871 airlines serving nearly 3.3 billion individuals, and the number of daily flights exceeded 100,000, so that a large-scale, dynamic and complex aviation network (AN) had been formed. As the prevalent intercontinental and intracontinental transportation system, AN affects many global issues like resource allocation^3^, forecasts of epidemics^4^, optimization of transportation systems^5^, etc.

Complex network theories have enabled us to quantify and understand the complexity and mechanisms behind the structure of AN^6, 7^. The results of an empirical analysis of the Worldwide Aviation Network (WAN) in 2002^8^ illustrated that WAN was a typical scale-free small-world network. To understand the weighted features of WAN, a spatial weighted model was proposed by Barrat *et al*.^9^. These authors studied the correlations between the weighted quantities and the topology, and the effects of space on centrality, clustering and assortativity. On the other hand, empirical studies of domestic aviation networks (e.g., in US (USAN)^10^, China (CAN)^11^ and India (IAN)^12^) reveal that they have structural properties that are different from those of WAN, such as a two-regime power law degree distribution^12^. In addition to topological analyses, others have studied issues including key nodes^13, 14^, flight delay^15–17^, the vulnerability of AN^18^, etc. To characterize the impact of an AN on epidemics, Hufnagel *et al*.^19^ proposed a probabilistic model to forecast the geographical spread of a worldwide disease. In a more recent study, Brockmann and Helbing^20^ successfully identified the spatial origins of the 2009 H1N1 and 2003 SARS epidemics using a passenger-flux motived distance. In addition, to reduce the contamination of disease with minimum interference with trade and travel, as International Health Regulations (IHR)^21^ calling for, screening of passengers in the airports involving outbreaks may play a critical role in hindering the spread of epidemics, especially during the incubation period^22–25^. Besides, efforts should be concentrated on travelers who are capable of effectively restraining the spread^26^ and international cooperations are essential to reduce global transmission^27^. Nevertheless, spreading models on static networks would produce exaggerated infection rates because it may result in large overestimation of the temporal duration of links and underestimation of distances between pairs of nodes^28, 29^; this has inspired recent studies on the impact of temporal patterns on network dynamics^30–32^.

However, it is not yet clear how the temporal structure of flights in an AN can change previous conclusions on the propagation process when the network is analyzed aggregately. To fill in this gap, in this study, we extract the duration and the temporal sequences of Chinese domestic flights, analyze CAN using a temporal approach^30, 33^ and run a Susceptible-Infectious (SI) disease spreading model^34^ to understand its time-respecting characteristics. We compare these measures to understand how the temporal information about the network complements the empirical evidence, and we show a temporal version of CAN. As the topological structure of CAN changes over time, an infectious individual can only infect its neighbors at a certain time, i.e., when they are connected. This mechanism is responsible for the slowing down of the transmission and is verified in our simulations.

## Results

### Static characteristics of topology

Our analysis involves a dataset for CAN in 2014, retrieved from the OK Traveling website^35^ which provides a complete list of all domestic flights information. The data comprises *N* = 183 airports as nodes and *L* = 14,268 scheduled flights as temporal links that connect pairs of airports. By aggregating the links on each route, we construct a static version of CAN with *E* = 1,627 weighted edges. As the first step, we investigate the degree distribution, *P*_*k*_, which describes the probability of an airport having *k* connections. The static CAN in this study reveals a scale-free behavior with a two-regime power law divided at *k* ^*^,

1 $${P}_{k}\sim \{\begin{array}{cc}{k}^{\alpha } & k\le {k}^{\ast }\\ {k}^{\beta } & k > {k}^{\ast }\end{array}$$

with *α* = −0.557(−0.610, −0.504), *β* = −2.408(−2.529, −2.289) under 95% confidence interval and *k* ^*^ = 30. At the same time, CAN shows small-world features with strengthened clustering (average clustering coefficient <*C*> = 0.73), short shortest path (average length <*l*> = 2.06) and more neighbors (average degree <*k*> = 17.8). From Table 1, in comparison to CANs described in 2005 and 2010, we can see that as a consequence of the increased network density, the current CAN has a higher average degree and clustering coefficient, and shorter average shortest path lengths. The addition of new airports with minor flights connecting hub airports may explain the distinction, and this also induces the increased heterogeneity among nodes observed from the decreasing exponents of the degree distribution.

**Table 1:** Comparison of topological characteristics of static CAN.

	*N*/*E*	*P*_*k*_	<*k*>	*C*	<*l*>
Liu *et al*.^36^	121/1378	*k* ^*^ = 20, *α* = −0.530, *β* = −2.050	11.38	0.75	2.26
Zeng *et al*.^37^	161/1185	*k* ^*^ = 29, *α* = −0.408, *β* = −2.166	14.72	0.70	2.14
This study (2014)	183/1627	*k* ^*^ = 30, *α* = −0.557, *β* = −2.408	17.80	0.73	2.06

### Distribution and dynamics of flights

**Figure 1:**
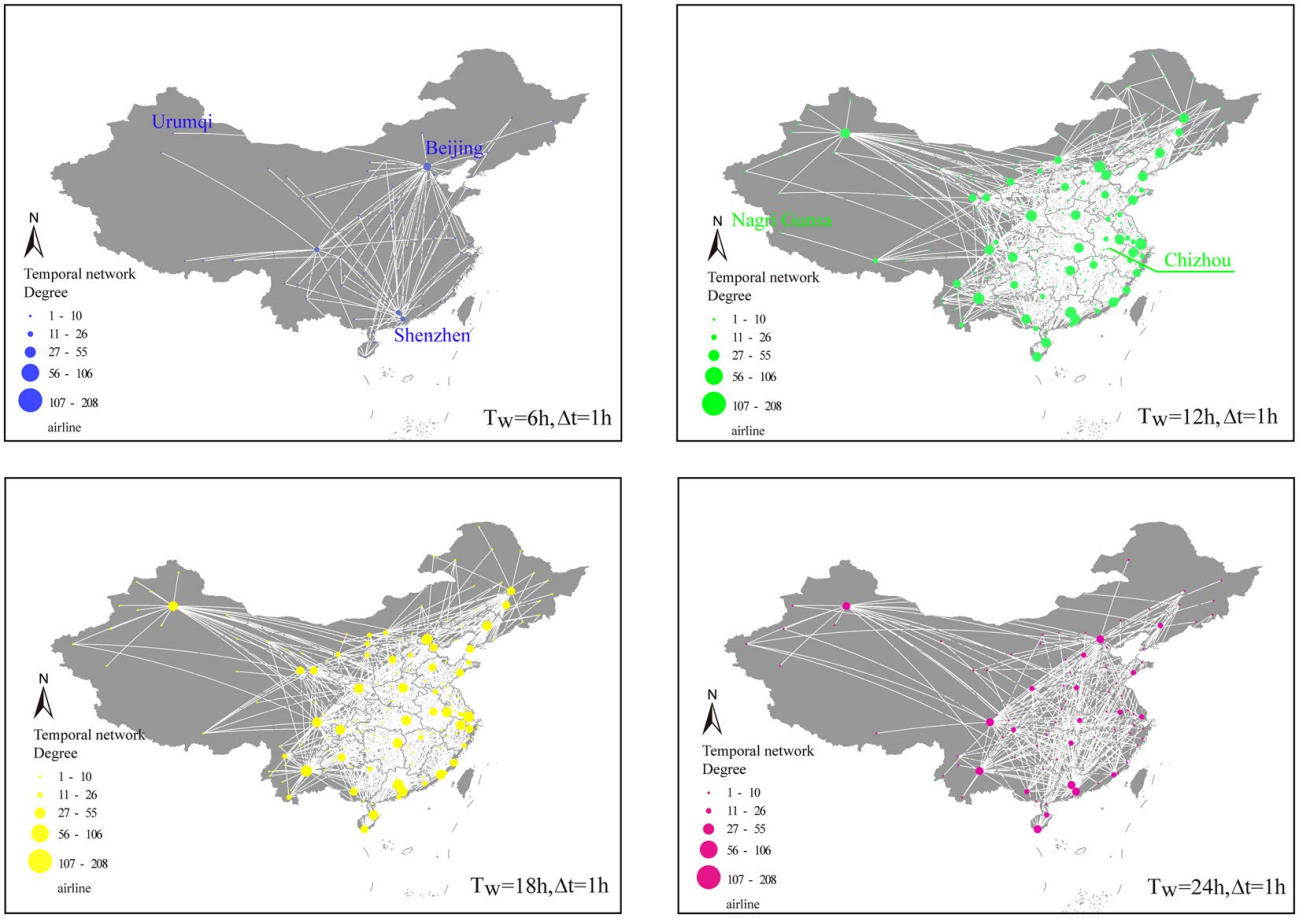
Temporal-spatial distribution of airports in CAN. Four slices of sub-network are shown. *T*_*w*_ represents the initial time of a given time window, and Δ*t* denotes the length of the time window. The map is obtained from OpenStreetMap.org under the Open Data Commons Open Database License (ODbL), and visualized with ArcGIS 10.0 (http://www.esri.com/).

The sequence of flights and the duration of each flight make it possible for us to investigate the temporal characteristics of CAN. In this study, we represent the temporal CAN during a day by quadruplets $(i,j,{t}_{ij}^{s},{t}_{ij}^{e})$ which describe where and when each flight starts and ends (see Methods). The edges between pairs of nodes appear at times ${t}_{ij}^{s}=\{{t}_{ij}^{s}(1),{t}_{ij}^{s}(2),\mathrm{...},{t}_{ij}^{s}(n)\}$, which are ordered such that ${t}_{ij}^{s}(a) < {t}_{ij}^{s}(b)$ if *a* < *b*, where *n* is the total number of flights on that edge. In addition, since we assume that edges establish when the flights on it start until the time the flights end, edges may be overlapped because of the duration of flights: flights on the same edge starting at different times may be present simultaneously at some times, and this can be illustrated as weights on edges during that time. We analyze the temporal characteristics of CAN by sliding CAN with an hourly time window. Each slice of CAN is represented as an aggregated sub-network as Fig. 1 shown.

**Figure 2:**
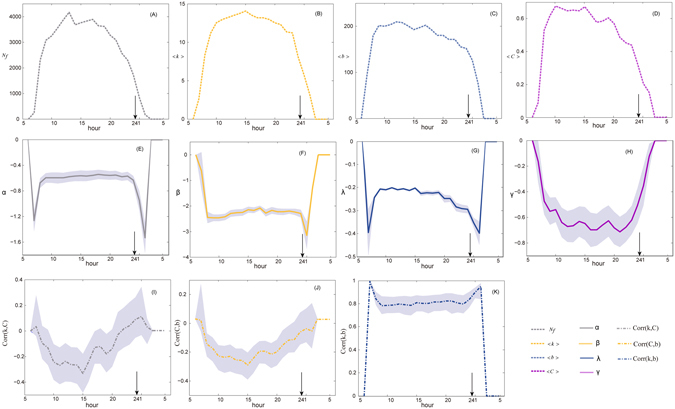
Temporal properties of the topology of CAN. 95% confidence interval are shown from (**E**) to (**K**). (**A**) Temporal property of the dynamics of flights. (**B**–**D**) Temporal properties of the averages of statistics. (**E**–**H**) Temporal properties of the distribution functions. *λ* and *γ* are parameters for the distributions of betweenness and the clustering coefficient, respectively, and *α* and *β* are parameters for the two-regime power law degree distribution. (**I**–**K**) Temporal properties of correlations between degree *k*, betweenness centrality *b* and clustering coefficient *C*. *Corr*(*x*, *y*) denotes the correlation between *x* and *y*.

We start analyzing the temporal traveling pattern by calculating the change in the number of flights (*N*_*f*_). As shown in Fig. 2A, the number starts from zero at 6:00, increases dramatically during the following three hours, and peaks at 13:00. It continues at high level until 18:00, then there is a relatively slight decrease and the number returns to zero from 3:00 to 6:00 as there are no flights during this time. 88.37% of all flights take place between 10:00 and midnight, and the peak hours are from 10:00 to 18:00, when 59.22% of all flights occur. These observations confirm our claim that the pattern of inter-city travel by air differs from that for daily commuting within a city by bus or car. The former has one long peak period, while the latter shows two obvious peaks at commuting times^38^.

The spatial variation and the evolution of connectivity for CAN are illustrated in Fig. 1. As we can see, the aggregated sub-network from 6:00 to 7:00 displays low connectivity and a sparsity of spatial distribution. Flights during this period are usually long-distance trips that take a long time (e.g. from Beijing to Urumqi) and connect airports with high travel demand (e.g. Beijing and Shenzhen). Shortly after the rapid increase in flights at noon, CAN experiences an impressive growth in the number of flights, resulting in high connectivity and spatial density, which can be explained from the perspective of the emergence of new links (especially those connecting remote cities) driven by the rapid rise in demand for air travel. After 18:00, most passengers have finished their journeys, reducing the demand for travel and relieving the transport pressure at many airports. In addition, several airports become silent before 18:00; this is especially true for airports located in sparsely-populated cities, such as Nagri Gunsa Airport, or those located in the neighbouring cities as hub airports, such as Chizhou Airport, which are used to ease the pressure on the hub at peak times. At the end of the day (at midnight), airports serving long-distance journeys are waiting for flights to land, thus contributing to the relatively higher connectivity at this time than at 6:00.

### Temporal characteristics of topology

Centrality is a common measure for identifying important nodes within a network. The centralization of an entire network is usually defined as the average of the nodal centrality. For example, the average degree centrality <*k*> shows the average number of connections involved at a node, the average betweenness centrality <*b*> indicates the average frequency at which a node is traversed by the shortest paths, and the average clustering coefficient <*C*> measures the average probability that “your friends’ friends are your friends”, or the ratio of triangles within the network from the perspective of topology. The time series of these measurements of centrality during the 24 sub-networks, which are presented in Fig. 2B–D, enable us to analyze the temporal characteristics of CAN. We can see that all of these measurements show a peak and an off-peak pattern which is similar to that observed for *N*_*f*_. Nevertheless, the growth during the dawn hours *P*_*dawn*_ (6:00 to 9:00) and the drop during the night hours *P*_*night*_ (22:00 to 3:00) are both at a faster rate, which implies that the small addition of flights during *P*_*dawn*_ may result in a significant increase in the centralization of CAN and the reduction of flights during *P*_*night*_ leads to a significant decline. Most flights during these periods are indispensable connections between pairs of nodes, and thus the changes for the connecting edges may have a great influence on the connectivity. In addition, the influence of the number of flights on topological statistics are different. The clustering coefficient decreases faster than that of degree and betweenness due to the reduction of flights.

As the average value is susceptible to extremes and skewed data distributions, we analyze the alteration of distribution functions that display the overall characteristics of the network. As mentioned before, the cumulative degree distribution of CAN follows a two-regime power law with parameters (see Formula 1) *α* and *β*, while the cumulative distributions of betweenness and the clustering coefficient follow *P*(*b*) ~ exp(*λ* · *b*) and *P*(*C*) ~ *γ* · *C*, respectively^39^. As shown in Fig. 2E–H, all parameters fluctuate dramatically during *P*_*dawn*_ and *P*_*night*_ because of the changes in essential connections. The relative stability of *α*, *β* during the remaining periods indicates that the majority of reductions and additions of flights occur along connections with multiple alternative flights. The gradual decrease of *λ* implies reductions in the critical edges on the shortest paths between pairs of nodes, whilst the cyclical change of *γ* illustrates that flights from or to airports with few connections, which are essential in enhancing the connectivity of CAN, are usually scheduled periodically.

Empirical studies on the static CAN have shown that high betweenness centrality for a node is usually associated with high degree, and that large clustering coefficient is usually associated with low degree^40^. However, such correlations may be different qualitatively and quantitatively when we consider the temporal effect of links. As shown in Fig. 2I–K, the relationship between degree and betweenness stays constant at about 0.8 during most intervals, but is higher during *P*_*dawn*_ and *P*_*night*_. The missing connections between locally-dominant airports in sparsely-populated districts and their subordinate airports may explain the high correlation. Airports with large betweenness but small degree on the aggregated version of the network, such as Urumqi, are usually central cities of remote districts and they form bridges connecting the local centers with political centers (e.g. Beijing) or economic centers (e.g. Shanghai). Interestingly, the clustering coefficient is positively related to the degree during *P*_*dawn*_ and *P*_*night*_, because airports operating during these periods usually serve individuals with high travel demand and most connections during that time are established among them. In the other intervals, possible connections among airports increase faster than the actual connections, and thus the degree climbs but the clustering coefficient decreases. In addition, the negative relationships between degree and clustering coefficient and betweenness and clustering coefficient show a rapidly increasing trend followed by a decreasing trend. The absence of small airports and the consequence that there are few links may be responsible for the subsequent decline.

### Classification of nodes based on burstiness

**Figure 3:**
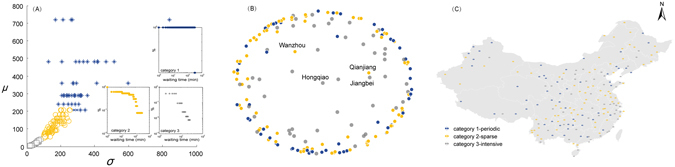
Airport classification based on burstiness. (**A**) Clustering based on the standard deviation (*σ*) and the mean (*μ*) of the waiting time between consecutive flights. Examples for each class are shown in the insets–Chengdu Shuangliu Airport for category one, Daqing Saertu Airport for category two and Kuqa Airport for category three. (**B**) Distribution of airports using a chart based on the GDP of the relevant cities. The center represents Hongqiao Airport and the radius denotes the gap between the GDP of Shanghai (the richest city in China) and other cities. (**C**) Spatial distribution of airports by category (mainland in China). The map is obtained from OpenStreetMap.org under the Open Data Commons Open Database License (ODbL), and visualized with ArcGIS 10.0 (http://www.esri.com/).

The dynamic behavior pattern of a temporal network can be quantified by burstiness, which is a measurement describing the phenomenon of a large number of events occurring in a short time and usually being followed by a long temporal gap before the next event. Burstiness is usually related to the standard deviation (*σ*) and the mean (*μ*) of the waiting time (see Methods) between consecutive events on the same airport^41^. To show the heterogeneity between nodes in terms of the behavior pattern, we classify the airports through a density-based clustering method, DBSCAN, with the radius *eps* = 50 and the minimum points within the radius required to form a cluster *Minpts* = 40 (see Methods). As shown in Fig. 3A, airports are classified into three categories. We characterize category one as “periodic”, as the distribution of waiting time can be expressed by several horizontal lines because there are only a few flights occurring at fixed time; category two as “sparse”, as we can fit the distribution of waiting time with a two-regime power law because of the several flights occurring with a long temporal gap (100 minutes or more); category three as “intensive”, as the distribution of waiting time can be fitted by a power law since there are a large number of flights and the gaps between flights are short (60 minutes or less).

Airports in different categories are identified in Fig. 3B, with Hongqiao Airport in Shanghai being the center, where the radius denotes the gap between the GDP of Shanghai (the richest city in China) and other cities. The map supports the assumption that the economy of neighbouring city (e.g., GDP) may be one of the dominant factors for the temporal characteristics of airports. As shown in Fig. 3B, the “intensive” cities mostly have high GDP, but airports in rich cities may be far from “intensive” if they are coexistent with the hub airport in this city. For example, Wanzhou, Qianjiang and Jiangbei airport in Chongqing playing different local roles show distinct temporal characteristics, thus belonging to two different categories. The spatial distribution of these categories (see Fig. 3C) illustrates that “intensive” airports are mainly located in coastal and capital cities where travel demand is rising quickly; the “sparse” airports are widely distributed and are mainly concentrated in the north-eastern and middle parts of China which are fiscally subordinate to the “intensive” cities; and the “periodic” airports are mainly located in the fiscally relatively poor cities as well as cities in between “intensive” and “sparse” airports.

### Spread of disease on temporal CAN

**Figure 4:**
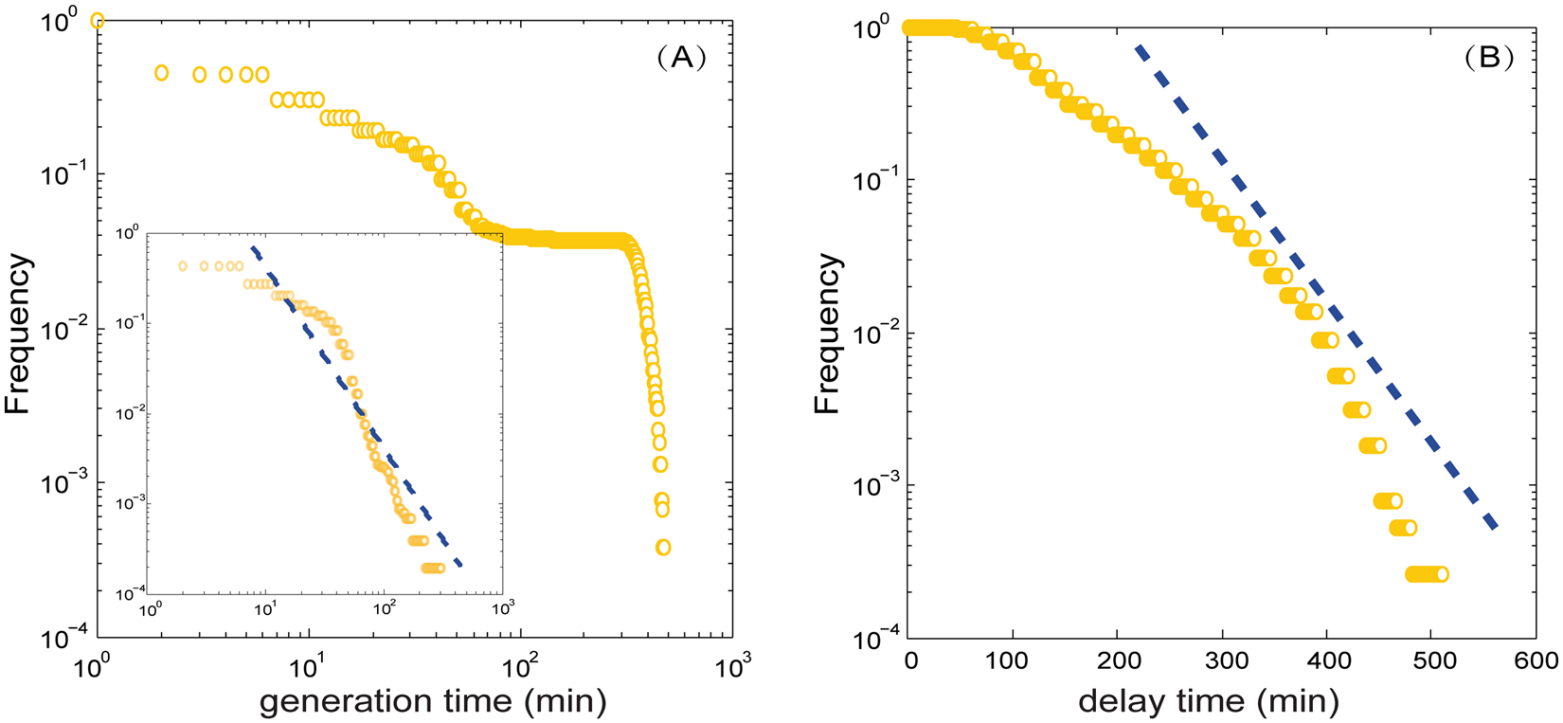
Distribution of generation time on nodes and delay time on edges within CAN. (**A**) Distribution of generation time on nodes. The distribution after 6:00 is shown in the inset. (**B**) Distribution of delay time on edges.

Studies on static networks claim that the network structure affects the speed and the reach of spreading through features^34^ like the degree distribution^42^, short path lengths^43^, or community^44^. However, recent studies^31, 32^ have shown that the sequential pattern of contacts plays a crucial role in spreading, and that biases may be introduced if temporal networks are treated aggregately when analyzing spreading dynamics. In Fig. 4, we show the distribution of the *generation time* on nodes (the temporal gap between the arrival and subsequent departure from the same airport) and the *delay time* on edges (the duration of flights) (see Methods). As we can see, there is a large gap in the generation time of approximately 100 minutes because of the silence of CAN between 3:00 and 6:00. Nevertheless, the distribution of the generation time follows a power law if we ignore the sub-networks before 6:00 (the mechanism we adopted for the Susceptible-Infectious spreading model). In addition, the delay time on edges follows an exponential distribution.

To understand the effects of generation time and delay time on spreading within CAN, we introduce two kinds of airport-oriented SI spreading model: the asynchronous SI spreading model (ASSI) and the synchronous SI spreading model (SSI). Nodes within both models belong to either susceptible (S) or infectious (I), and S may with probability *α* be infected by its infectious neighbors (see Methods). However, the time when the infection completes and the duration of status of nodes are different. Events (flights) within ASSI are represented as a sequence of triples $(i,j,{t}_{ij}^{e})$ by considering the temporal delay of completion of infection. The infection in ASSI is completed at the time when the flight lands $({t}_{ij}^{e})$ and the susceptible node will be infected if its neighbours are infectious at ${t}_{ij}^{e}$. On the other hand, events on SSI are treated as a sequence of quadruplets $(i,j,{t}_{ij}^{s},{t}_{ij}^{e})$, ignoring the temporal delay caused by the delay time on edges. The infection in SSI is completed when the flight starts $({t}_{ij}^{s})$ and the status of nodes continues until the flight ends $({t}_{ij}^{e})$, and the susceptible node will be infected if its neighbours are ever infectious during $({t}_{ij}^{s})$ and $({t}_{ij}^{e})$. We can see that ASSI is able to simulate the process of infection with temporal duration, such as the propagation of worldwide diseases among cities, while SSI is a man-made comparative model without any representations in reality to study the effect on spreading of the delay time on edges. In the following, we run these models together with classical aggregated SI spreading model (ASI) by setting *α* = 0.5.

**Figure 5:**
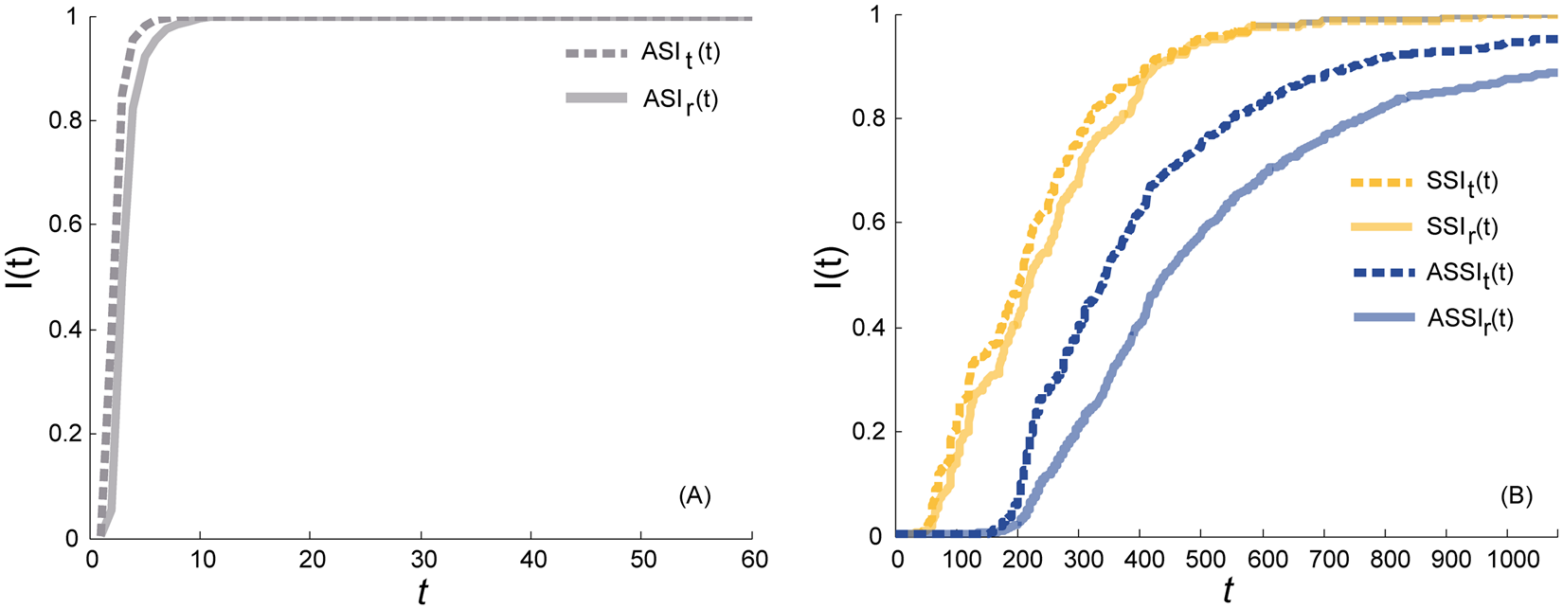
Comparison of propagation patterns within different SI spreading models on CAN. (**A**) Spreading pattern in ASI. *ASI*_*t*_(*t*) and *ASI*_*r*_(*t*) denote the ratios of infected nodes in ASI at time *t* in the case of targeted and random infections, respectively. (**B**) Spreading patterns in ASSI and SSI. *SSI*_*t*_(*t*), *SSI*_*r*_(*t*), *ASSI*_*t*_(*t*), *ASSI*_*r*_(*t*) denote the ratios of the infected nodes in SSI and ASSI at time *t* in the case of targeted and random infections, respectively.

As shown in Fig. 5, spreading of ASSI and SSI is much slower than that in ASI no matter where it originates, illustrating that the generation time on nodes is the main contributor to the slowing down of the spread, which echoes the conclusion in previous study^32^. Besides, propagation starts much later in SSI and ASSI than that in ASI, showing that temporal sequence of links between airports inhibits the outbreak of spreading, while the temporal gap between outbreaks in ASSI and SSI is a consequence of the lack of delay time on edges. In addition, not all nodes will be infected at the end of a day within ASSI because flights involving susceptible airports at that time complete at the next day. Moreover, by changing the infection sources, we find that propagation originating from airports with the largest degree (targeted infection) is faster than that from random nodes (random infection) for all SI spreading models on CAN, while targeted infection within ASSI enhances the speed and reach of spreading (see Fig. 5B), which is never examined before. This difference supports the claim that the duration on edges makes the temporal network more sensitive to targeted infection.

## Discussion

In summary, using the Chinese Aviation Network (CAN) data in 2014, we find that the aggregated CAN in this study is more significant in terms of scale-free and small-world properties than CANs used in previous studies. The traveling pattern between cities, as reflected by the flights, is slightly different from the patterns for traveling by other means within metropolitan cities^38^, with a longer duration of peak hours. In the past, researchers^37^ have shown a negative correlation between degree and clustering coefficient within CAN. However, when we take temporal sequence of flights into account, a positive correlation of these two indices is discovered for the time interval between 22:00 and 2:00 on the next day. In addition, flights during the emergence of CAN (from 6:00 to 9:00) and at night (22:00 to 3:00) may result in a significant decrease in the centralization of the network, because most of them are essential connections between airports. The temporal characteristics of an airport are mainly relevant to the economy of the neighbouring city and to the role it plays in air transportation within this city. Interestingly, the temporal sparsity of the generation time slows down the spread on CAN, while the duration of flights enhances the sensitiveness of CAN to the targeted infection.

The above findings not only reveal specific topological and temporal structural pattern for CAN, but also provide insights for the study of other local or global aviation networks. Although it might be different for network structure and temporal characteristics of airports, such as temporal-spatial distribution of airports, temporal properties of the topology, etc. Nevertheless, comparing with WAN where some airports are always in daylight due to the rotation of the earth, conclusions irrelevant to the temporal span may be similar, e.g., the dominant role of GDP of belonging cities in shaping the temporal pattern of airports, the slower spreading on temporal networks than that on static networks, etc. For other local aviation networks, there may be more similarities including the characteristics of the sequence of sub-networks in one day, the shift from negative to positive correlation between clustering coefficient and degree, etc.

The research about temporal networks is a new and booming field^30^, and existing works mainly concern theoretical developments^45–47^. Our study is not only an important supplement to the analysis of static aviation networks, but also a successful application of temporal network theory to uncover the general spreading patterns on modern infrastructure systems. The results are therefore crucial for further understanding of aviation networks and the dynamic processes affected by them, such as cascading failures for flight delays^15^, the movement of populations and the spread of diseases^4^.

Since the continuous time on temporal CAN is discretized by the time window in the SI model, some temporal characteristics involving the whole interval such as reachability^30^ may be missing and that more complicated epidemiology models may be examined. Moreover, the effect of other temporal and topological characteristics, e.g., weighted degree^48^, optimal path of airline^49^, is to be investigated in further research.

## Methods

### Chinese Aviation Network (CAN) data

We retrieve all flight schedules within mainland in China during the spring and summer of 2014 from the most comprehensive flight-oriented traveling website–OK traveling website^35^. The raw data includes start and end time of each flight, depart and arrival airports, as well as geo-locations of each airport. Data are available for 1,627 domestic routes and 14,268 scheduled flights operated by 28 airline companies in China, including Southern Airlines, Xiamen Airlines, Air China and etc. In addition, the data contains a few circular flights which go from A to C through B and then return to A without going through B. Since the circular flights are few and usually involve remote cities in China, we consider these as unidirectional flights in opposite directions.

The layout of CAN is generated using the longitude and latitude of each airport. We embedded the airports in a two dimensional space using a rectangular projection of the Earth. The edges are placed between pairs of airports if there is a direct flight connecting them during a given time interval.

### Time Window

We represent CAN observed during a particular time interval by a contact sequence of quadruplets $(i,j,{t}_{ij}^{s},{t}_{ij}^{e})$, where *i, j* denote the airports and ${t}_{ij}^{s},{t}_{ij}^{e}$ are the take-off and landing times of the flight^30^. In this study, we assume that it is not the nodes but the connections from or to them that change over time, i.e. *e*_*ij*_(*t*) = 1 if ${t}_{ij}^{s}\le t\le {t}_{ij}^{e}$, otherwise *e*_*ij*_(*t*) = 0. Based on this assumption, we partition the daily CAN into consecutive sub-networks, with each network being constructed of flights with *e*_*ij*_(*t*) = 1 during that time window^50^. The sub-network contains the departing, landing and ongoing flights as following,

2 $$\{\begin{array}{l}{T}_{w}(m) < {t}_{ij}^{s}\le {T}_{w}(m)+{\rm{\Delta }}t\\ {T}_{w}(m) < {t}_{ij}^{e}\le {T}_{w}(m)+{\rm{\Delta }}t\\ {t}_{ij}^{s}\le {T}_{w}(m)\,and\,{t}_{ij}^{e} > {T}_{w}(m)+{\rm{\Delta }}t\end{array}$$

where *T*_*w*_(*m*) represents the initial time of the *m* th time window, and Δ*t* denotes the length of the time window or the temporal resolution of CAN and is set as Δ*t* = 1*h* in this study.

### Classification based on burstiness

To study the similarity and dissimilarity of airports in terms of their temporal characteristics, we classify them based on their behavior patterns, which can be represented by their burstiness–a quantitative measurement of how bursty they are, and can be defined as a function of the mean (*μ*) and standard deviation (*σ*) of the waiting time (*W*_*t*_)^51^. The waiting time between two consecutive events at a given node, say $(i,j,{t}_{ij}^{s},{t}_{ij}^{e})$ and $(i,k,{t}_{ik}^{s},{t}_{ik}^{e})$ on node *i* where ${t}_{ik}^{s}\ge {t}_{ij}^{s}$, is ${W}_{t}={t}_{ik}^{s}-{t}_{ij}^{s}$. We embedded the nodes in a two dimensional space using the two parameters of burstiness, and detect possible categories through Density-based Spatial Clustering of Applications with Noise (DBSCAN)^52^ which is robust to outliers. This method requires two priori inputs–the radius (*eps*) and the minimum points (*MinPts*) within the radius required to form a dense cluster. If the number of points within the radius of an unvisited point exceeds *MinPts*, the unvisited point will be a part of the cluster which contains the point whose radius covers it, and otherwise it is noise.

### SI spreading models

To begin with, we formulate the generation time on nodes (*G*_*t*_) and delay time on edges (*D*_*t*_). The generation time of two flights on nodes, say $(i,j,{t}_{ij}^{s},{t}_{ij}^{e})$ and $(j,k,{t}_{jk}^{s},{t}_{jk}^{e})$ on node *j* where ${t}_{jk}^{s} > {t}_{ij}^{e}$, is ${G}_{t}={t}_{jk}^{s}-{t}_{ij}^{e}$, while the delay time on edges, say $(i,j,{t}_{ij}^{s},{t}_{ij}^{e})$, is defined as ${D}_{t}={t}_{ij}^{e}-{t}_{ij}^{s}$.

To figure out the effects on spreading of generation time and delay time, we introduce two kinds of airport-oriented SI spreading models: the asynchronous SI spreading model (ASSI) and the synchronous SI spreading model (SSI). Infections in SSI complete at the moment when the flight starts and the status of nodes continues until the moment when the flight ends, i.e. ${e}_{ij}(t)=1,{t}_{ij}^{s}\le t\le {t}_{ij}^{e}$ from the perspective of network, while infections in ASSI are complete when the flight ends, i.e. ${e}_{ij}({t}_{ij}^{e})=1$. Then, we run these models along with the classical aggregated SI spreading model (ASI) to evaluate and compare the difference in propagation patterns. In all versions of SI spreading model, nodes belong to one of two categories: susceptible (S) and infectious (I). In addition, S may with probability *α* be infected by its infectious neighbors without recovering.

In ASSI, infections begin when flights start and complete when flights end, which means that nodes can be infected if their neighbours are infectious at the departure time of flights, i.e. ${I}_{{t}_{ij}^{e}+1}={I}_{{t}_{ij}^{e}}+\alpha {S}_{{t}_{ij}^{s}}$. In SSI, infections complete when flights start, i.e. ${I}_{{t}_{ij}^{s}+1}={I}_{{t}_{ij}^{s}}+\alpha {S}_{{t}_{ij}^{s}}$. In ASI, infections occur at any time, i.e. ${I}_{t+1}={I}_{t}+\alpha {S}_{t}$. The fundamental cause of the difference between the process of propagation focus on the infectious rate *α* which is changeable with the connections between airports *e*_*ij*_. In ASI, *α* keeps a constant since *e*_*ij*_ never change, while in temporal SI spreading models, it depends on the connection between airports, i.e.,

3 $$\{\begin{array}{cc}\alpha =0 & {e}_{ij}(t)=0\\ \alpha =C & otherwise\end{array}$$

where *C* is a constant. Then, we can rewrite the propagation process as following which suits all SI spreading models in this study,

4 $$\{\begin{array}{rcl}{I}_{t+1} & = & {I}_{t}+\alpha {S}_{t}\\ {S}_{t+1} & = & 1-{I}_{t+1}\end{array}$$
